# Identification of an imidazoline binding protein: Creatine kinase and an imidazoline-2 binding site

**DOI:** 10.1016/j.brainres.2009.04.044

**Published:** 2009-07-07

**Authors:** Atsuko Kimura, Robin J. Tyacke, James J. Robinson, Stephen M. Husbands, Michael C.W. Minchin, David J. Nutt, Alan L. Hudson

**Affiliations:** a Psychopharmacology Unit, University of Bristol, BS1 3NY, UK; b Department of Pharmacy and Pharmacology, University of Bath, BA2 7AY, UK; c Astellas Pharma Europe Ltd., Staines, Middlesex TW18 3AZ, UK; d Department of Pharmacology, 9-70 Medical Sciences Building, University of Alberta, Edmonton, Alberta, Canada T6G 2H7

**Keywords:** 2-BFI, 2-(2-benzofuranyl)2-imidazoline, BU224, 2-(4,5-dihydroimidaz-2-yl)quinoline, BU99006, 5-isothiocyanoato-2-benzofuranyl-2-imidazoline, B-CK, brain creatine kinase, CK, creatine kinase, GOLD, genetic optimisation for ligand docking, GR, glucose-responsive, I_2_, imidazoline-2 subtype, K_ATP_ channel, ATP sensitive potassium channel, MAO, monoamine oxidase, MOE, molecular operating environment, Imidazoline binding protein, Creatine kinase, 2-BFI, Harmane and psychiatric disorders

## Abstract

Drugs that bind to imidazoline binding proteins have major physiological actions. To date, three subtypes of such proteins, I_1_, I_2_ and I_3_, have been proposed, although characterisations of these binding proteins are lacking. I_2_ binding sites are found throughout the brain, particularly dense in the arcuate nucleus of the hypothalamus. Selective I_2_ ligands demonstrate antidepressant-like activity and the identity of the proteins that respond to such ligands remained unknown until now. Here we report the isolation of a ∼ 45 kDa imidazoline binding protein from rabbit and rat brain using a high affinity ligand for the I_2_ subtype, 2-BFI, to generate an affinity column. Following protein sequencing of the isolated ∼ 45 kDa imidazoline binding protein, we identified it to be brain creatine kinase (B-CK). B-CK shows high binding capacity to selective I_2_ ligands; [^3^H]-2-BFI (5 nM) specifically bound to B-CK (2330 ± 815 fmol mg protein^− 1^). We predicted an I_2_ binding pocket near the active site of B-CK using molecular modelling. Furthermore, B-CK activity was inhibited by a selective I_2_ irreversible ligand, where 20 μM BU99006 reduced the enzyme activity by 16%, confirming the interaction between B-CK and the I_2_ ligand. In summary, we have identified B-CK to be the ∼ 45 kDa imidazoline binding protein and we have demonstrated the existence of an I_2_ binding site within this enzyme. The importance of B-CK in regulating neuronal activity and neurotransmitter release may well explain the various actions of I_2_ ligands in brain and the alterations in densities of I_2_ binding sites in psychiatric disorders.

## 1 Introduction

The existence of imidazoline binding sites has been established and their functions together with underlying mechanisms have been debated for several years. Currently, there are three defined subtypes for these sites. The clonidine preferring I_1_ site is found in the brainstem and is associated with blood pressure control (Bousquet et al., 1984). The I_2_ sites have been identified using idazoxan and these are predominantly found in the brain and liver where they regulate monoamine turnover (Alemany et al., 1997). The I_3_ sites are located in pancreatic β-cells and these regulate insulin secretion (Chan et al., 1994). The physiological relevance of these sites is strengthened by the findings that endogenous substances especially agmatine (Li et al., 1994) and harmane (Hudson et al., 2001) have significant activity at one or more of these sites. Furthermore, ligands targeting these sites demonstrate therapeutic potentials in psychiatric disorders (Smith et al., 2009).

**Fig. 1:**
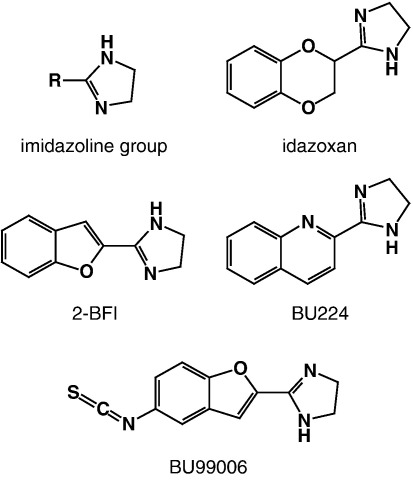
Chemical structures of selective I_2_ ligands.

In order to advance our knowledge of properties of I_2_ binding proteins, several selective ligands were synthesised including 2-(2-benzofuranyl)2-imidazoline (2-BFI), 2-(4,5-dihydroimidaz-2-yl)quinoline (BU224) and an irreversible ligand 5-isothiocyanoato-2-benzofuranyl-2-imidazoline (BU99006) (Fig. 1). These ligands have established the distribution of I_2_ sites; most interestingly, the density of I_2_ sites is extremely high in the arcuate nucleus in rat brain (Lione et al., 1998).

Understanding the nature of I_2_ sites is important for a number of reasons. Ligands selective for I_2_ sites have been shown to regulate the release of biogenic amines such as dopamine and noradrenaline and probably through these mechanisms, they demonstrate antidepressant-like properties (Finn et al., 2003). Furthermore, post-mortem studies have shown that the density of I_2_ sites was altered in suicide/depression and increased in Alzheimer's disease (García-Sevilla et al., 1996, 1998), whereas there was a profound loss of these sites in the basal ganglia of patients with Huntington's disease (Reynolds et al., 1996). Moreover, brain I_2_ sites are down-regulated in opiate dependence both in rat and human (Hudson et al., 1996; Sastre et al., 1996). In addition, selective I_2_ ligands promote food intake (Nutt et al., 1995), which is presumed to be an effect through the arcuate nucleus of the hypothalamus since this brain region has a markedly high density of I_2_ sites (Lione et al., 1998).

The identity of imidazoline binding proteins has attracted interests of many research groups; however, determining its identity has proven challenging. It is only recently that an imidazoline binding protein of the I_1_-subtype was proposed to be a receptor that belongs to the sphingosine-1-phosphate (S1P)-receptor family (Molderings et al., 2007). For the I_2_-subtype, the high abundance of I_2_ sites in liver led to an exploration of monoamine oxidase as the possible I_2_ binding protein over a decade ago. Subsequently, it was reported that both monoamine oxidase (MAO) isoenzymes (A and B) show a low affinity binding site for I_2_ ligands (Parini et al., 1996) and that the nature of interaction was competitive (Ozaita et al., 1997; Jones et al., 2007). However, the distribution of I_2_ site is not contiguous with the distribution of MAO A or B (Eglen et al., 1998) and discrepancies in the pharmacological profiles regarding the interaction between MAO and I_2_ ligands (Ozaita et al., 1997) indicated that there must be other proteins that contain I_2_ binding domains. To date, a number of I_2_ binding proteins have successfully been isolated and their existence as a heterogeneous population has strongly been suggested (Escribá et al., 1999). However, a definitive I_2_ binding protein has yet to be identified; although, a ∼ 45 kDa protein has previously been isolated from rat brain (Escribá et al., 1995) and other studies have shown the expression of this protein is altered in Alzheimer's disease and depression (García-Sevilla et al., 1996, 1998).

The aim of this study was to isolate the ∼ 45 kDa binding protein from rabbit and rat brain, and determine its identity. We utilised a highly selective I_2_ ligand, 2-BFI, as a key tool in affinity chromatography and employed N-terminal protein sequencing to reveal the identity of the isolated protein. Subsequently, we confirmed properties of this protein as an I_2_ binding protein using biochemical and pharmacological techniques.

## 2 Results

### 2.1 Isolation of I_2_ binding proteins using 2-BFI affinity column

**Fig. 2:**
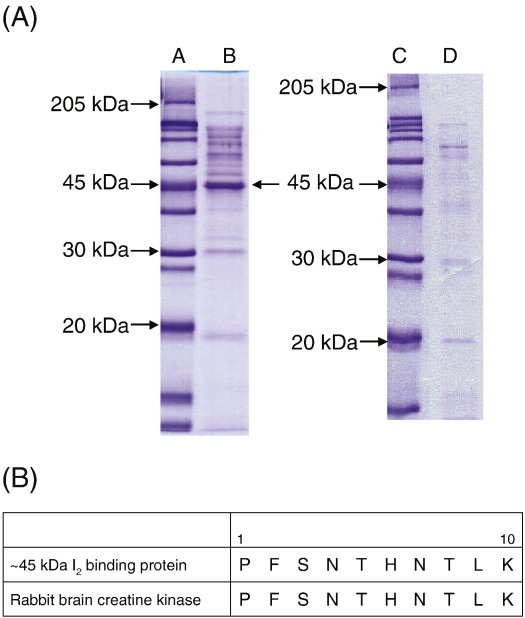
(A) I_2_ binding proteins from rabbit brain isolated using a 2-BFI affinity column (lane B), molecular markers (lanes A and C) and proteins eluted from the control (blank) column (D), in which 2-BFI link was absent. Proteins were separated by SDS-PAGE (12%) and gels were stained with Coomassie Blue. As expected, a strong band at  ~45 kDa and a few other weaker bands were detected. This  ~45 kDa protein was absent from proteins eluted from the control column indicating that it specifically bound to the 2-BFI affinity column. The proteins eluted from the 2-BFI affinity column were transferred onto PVDF membranes and the  ~45 kDa band was subjected to protein sequencing. (B) Complete match of N-terminal protein sequences (10 amino acids) of the  ~45 kDa I_2_ binding protein and rabbit brain creatine kinase (EC 2.7.3.2). (For interpretation of the references to colour in this figure legend, the reader is referred to the web version of this article.)

Rabbit or rat brain I_2_ binding proteins were solubilised, loaded onto the 2-BFI affinity column and then eluted off using idazoxan (20 mM). The isolated proteins were concentrated and subjected to SDS-PAGE analysis for further separation according to their molecular weight. Coomassie Blue staining revealed a strong band at ∼ 45 kDa together with several weaker bands of various sizes (Fig. 2). This ∼ 45 kDa band was absent from the control column, in which 2-BFI was not linked to the matrix, indicating specific binding of this protein to 2-BFI (Fig. 2). The proteins separated by SDS-PAGE analysis were transferred onto PVDF membranes. The ∼ 45 kDa band was excised and subjected to N-terminal sequencing. The resulting peptide contained a ten amino acid sequence of -PFSNTHNTLK-. This sequence was used to search for the identity of this protein in a protein sequence database (Protein Information Resources, Wu et al., 2002). The exact match for the ten amino acid sequence of rabbit brain origin was confirmed as rabbit brain creatine kinase (B-CK, 43 kDa, EC 2.7.3.2); the match was found at the N-terminus of this enzyme. Extensive search of the identified peptide in protein sequence databases revealed no other possible candidate proteins. For rat brain, a BLAST search programme revealed the most probable protein for this isolated protein (∼ 45 kDa) to be rat brain creatine kinase.

### 2.2 [^3^H]-2-BFI binding to B-CK

**Fig. 3:**
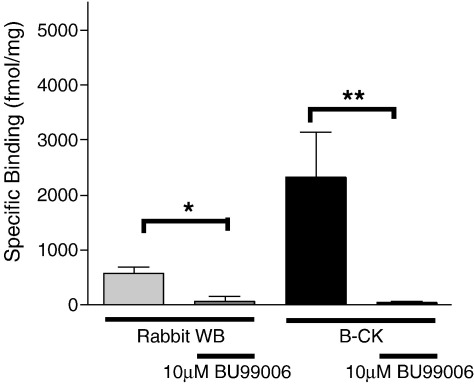
Specific binding of [^3^H]-2-BFI to rabbit brain creatine kinase (B-CK) and rabbit whole brain membranes (rabbit WB; control) with or without BU99006 (10 μM) pre-treatment. The specific binding to the control (568.8 ± 116.5 fmol mg protein^− 1^, *n* = 9) and B-CK (2329.5 ± 814.8 fmol mg protein^− 1^, *n* = 7) was abolished after pre-treatment with BU99006 (Student's *t*-test, *P < 0.04 and **P < 0.03 respectively, *n* = 4). These data represent mean ± SEM, *n* = 4–9.

Radioligand binding centrifugation assay was performed using [^3^H]-2-BFI (5 nM) in order to determine and compare the I_2_ binding capacity of purified rabbit B-CK and rabbit whole brain P2 membrane preparation. Rabbit B-CK was able to specifically bind to [^3^H]-2-BFI (2330 ± 815 fmol mg protein^− 1^, *n* = 7), where the specific binding was determined using another selective I_2_ ligand, BU224 (10 μM) (Fig. 3). These data indicate specific binding of the two selective I_2_ ligands to an apparent I_2_ site on this enzyme. The specific I_2_ binding was approximately 4 fold higher in B-CK than in rabbit whole brain P_2_ membrane. Pre-treatment with the irreversible I_2_ ligand, BU99006 (10 μM), significantly attenuated the specific I_2_ binding in both rabbit B-CK and rabbit whole brain P_2_ membranes (Student's *t*-test, *P* < 0.04 and *P* < 0.03 respectively), indicating that I_2_ sites occupied by [^3^H]-2-BFI were displaced by BU99006 due to its affinity also at the I_2_ sites. These data strongly support the existence of an I_2_ binding site on B-CK.

### 2.3 Effects of I_2_ ligands on B-CK activity

**Fig. 4:**
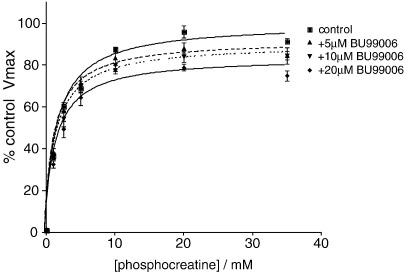
Dose-dependent inhibition of B-CK activity by BU99006 (5, 10 and 20 μM; pre-incubation), using phosphocreatine (0–35 mM) as the substrate. The data show mean percentage control to V_max_ ± SEM, *n* = 4.

The activity of B-CK was measured in the presence or absence of I_2_ ligands (1–20 μM; pre-incubation) using phosphocreatine (0–35 mM) as the substrate. There were no apparent changes in B-CK activity in the presence of 2-BFI, BU224, harmane, agmatine or amiloride, all of which have been reported to have affinity at I_2_ sites (data not shown). However, BU99006 inhibited the B-CK activity in a concentration-dependent manner, with the reductions in the apparent V_max_ by 8%, 10% and 16% at 5, 10 and 20 μM, respectively. These data indicate that the inhibition of B-CK activity by BU99006 at the tested concentrations is non-competitive (Fig. 4), which is consistent with the pharmacological profile of this compound determined previously (Tyacke et al., 2002).

### 2.4 Molecular modelling of I_2_ ligands docking to B-CK crystal structure

Currently, the only B-CK crystal structure available is that from chicken (Protein Data Bank ID (PDB): 1QH4). Chicken B-CK demonstrates ∼ 90% homology with rat and rabbit brain B-CKs, and thus this was considered suitable for use in our molecular modelling studies. The I_2_ binding domain on B-CK was predicted by molecular modelling using the SiteFinder option within MOE. Outside the active site, the top ranking binding site of chicken B-CK was a pocket adjacent to the active site encompassing amino acid residues Thr71, Val72, Val75, Leu201, Leu202, Cys283 and Ser285 amongst others.

**Fig. 5:**
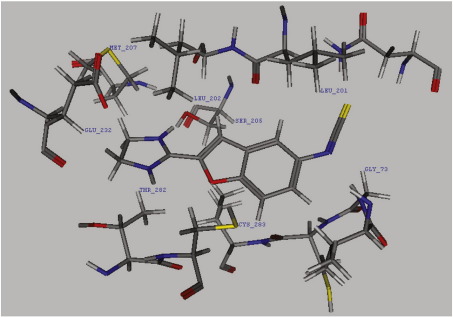
BU99006 docking to a crystal structure of the catalytic site of chicken brain creatine kinase (PDB: 1QH4). Amino acid residues in proximity to BU99006 (centre) were labelled including Cys283 (bottom centre). The isothiocyanate group on BU99006 is projected away from Cys283 in this illustration but the dynamic situations in vivo would allow the BU99006 to flip, making it within the reach of Cys283 forming a covalent bond. Also, it is possible that the covalent bonds are formed during the entry or exit of BU99006 to the binding pocket. 2-BFI, which shares the same structure as BU99006 apart from the isothiocyanate group, reversibly binds to this same pocket.

GOLD was utilised to predict a docking mode for both 2-BFI and BU99006, in their protonated form, in the site encompassing Cys283 defined by MOE. Both structures docked readily to this site (illustrated for BU99006 in Fig. 5). The proximity of the 2-BFI and BU99006 structures to Cys283 suggests that this nucleophilic residue could be responsible for the covalent interaction with BU99006. Covalent attachment of BU99006 to Cys283, followed by minimization, led to partial occupation of the enzyme active site (data not shown), which could explain the results from the pharmacological studies where this compound inhibited B-CK activity.

## 3 Discussion

The present study reports the identification of a ∼ 45 kDa imidazoline binding protein as brain creatine kinase (B-CK, 43 kDa, EC 2.7.3.2); this success was made possible largely due to the use of an affinity column generated with a highly selective I_2_ ligand, 2-BFI. This finding was confirmed in both rabbit and rat, the species most intensively studied for I_2_ binding sites. We demonstrated that B-CK showed a high I_2_ binding capacity and this specific binding was abolished by pre-treatment with an irreversible I_2_ selective ligand, BU99006. The activity of B-CK was inhibited by BU99006 and the molecular modelling predicted, with high probability, a binding pocket adjacent to the active site of B-CK. Our data clearly demonstrate the existence of an I_2_ binding site on B-CK.

Creatine kinase (CK) is a well characterised enzyme that is particularly important for energy homeostasis in cells. Briefly, CK transfers a phosphoryl group between ADP and ATP using creatine as an intermediate; the peptide sequence of CK across all species is highly conserved with ∼ 60% homology. There are two cytosolic isoenzymes, brain (B-) and muscle (M-) CK that are active in mono- or heterodimers and there are a further two mitochondrial octameric isoenzymes. Cytosolic B-CK converts ADP to ATP using phosphocreatine at sites of high energy demand and also converts ATP to phosphocreatine for energy storage, whereas mitochondrial creatine kinase preferentially uses ATP synthesised from oxidative phosphorylation to convert creatine to phosphocreatine and exports this to cytoplasm (for a review see Wallimann and Hemmer, 1994). B-CK is a cytosolic enzyme, but it is often tightly associated to synaptic vesicles (Lerner and Friedhoff, 1980) or directly and tightly coupled with membrane bound Na^+^/K^+^-ATPase (Blum et al., 1991) and also, CK has previously been reported to modulate neurotransmitter release (Dunant et al., 1988). Therefore, it is possible that a population of B-CK, which is bound to synaptic vesicles or to plasma membranes in neurons, is involved in neurotransmitter release as well as in the maintenance of membrane potential through the Na+/K+ ATPase. Such a population of membrane associated B-CK may explain how B-CK, typically a cytosolic soluble protein, remained in our P_2_ membrane preparation. There is evidence that I_2_ sites are found in plasma membranes (Heemskerk et al., 1998) and that I_2_ ligands elevate monoamine release (Nutt et al., 1995). To this end, further studies are required to investigate whether or not I_2_ ligands modulate neurotransmitter release through B-CK.

It was clear from the present study that B-CK contained a binding site for 2-BFI and the concentration of 2-BFI used has previously been shown to saturate a high component of I_2_ sites in rabbit brain (Lione et al., 1996). Under these assay conditions one could propose [^3^H]-2-BFI specifically bound to B-CK with apparent high affinity. This specific binding was abolished when B-CK was pre-treated with an irreversible I_2_ ligand, BU99006, further indicating the existence of an I_2_ binding domain in this enzyme.

The results from the molecular modelling studies suggest that 2-BFI binds to B-CK in a cleft or a binding pocket adjacent to the active site. Access to this pocket does not appear to be a requirement for the catalytic activity of the enzyme suggesting that 2-BFI may occupy this site without interfering or affecting the activity of B-CK. This could explain the results of the radioligand binding assay and enzyme activity assay, in which only the irreversible ligand (BU99006) inhibited B-CK activity; reversible I_2_ ligands with high affinity at I_2_ sites tested in this study had no effect on B-CK activity. While docking studies indicate that BU99006 is able to reversibly occupy this cleft, results from pharmacological assays demonstrate that it irreversibly binds to and inhibits B-CK. The most suitable nucleophile located near the preferred binding pocket is Cys283. This residue is highly reactive and is conserved in all known peptide sequences of CK. The importance of Cys283 in enzyme activity was heavily debated until site-directed mutagenesis studies showed it plays a role in substrate binding synergism but not directly involved in catalysis (Furter et al., 1993). It is possible that BU99006 acts as an effective enzyme inhibitor by covalently binding to this residue and thus disturbing the synergism. In addition, the modelling studies indicate that the preferred binding mode for BU99006 upon covalent attachment results in partial occupation of the enzyme active site, which could be another explanation for the inhibitory effect.

The contradiction between the high affinity of reversible I_2_ ligands at I_2_ sites and lack of effects on the B-CK activity draws attention. We propose below the possible and likely explanations for the discrepancies observed in this study. Firstly, as described above, our molecular modelling predicted the existence of an I_2_ site within B-CK adjacent to the active site, and that the covalent attachment of BU99006 at the reactive Cys283 leads to a partial occupation of the active site. It is possible that, without the covalent attachment and the resulting partial occupation of the active site, an I_2_ ligand does not act as an effective enzyme inhibitor at the concentration tested (20 μM). Secondly, the discrepancies may have arisen because the concentration of the reversible (competitive) I_2_ ligands used in the assay was too low relative to the concentration of the substrate; and thus the reversible I_2_ ligands were unable to compete with the high concentration of the substrate (μM against mM). Nevertheless, B-CK clearly presents a functional target for BU99006 and further studies are required to confirm its functional interaction with other I_2_ ligands.

Taken together, our data demonstrate that B-CK is an imidazoline binding protein that contains an I_2_ binding site. However, there are likely to be other proteins with I_2_ binding sites in brain since the distribution pattern of I_2_ sites does not completely mirror that of B-CK (Lione et al., 1998; Sistermans et al., 1995).

How does the expression profile of ∼ 45 kDa imidazoline binding protein compare with that of B-CK? In some neurodegenerative disorders changes in expression levels of B-CK and I_2_ binding proteins are independently observed. For example, in Alzheimer's disease, a membrane associated CK fraction is increased (David et al., 1998), which is consistent with the increased imidazoline binding protein of ∼ 45 kDa detected by immunoblotting in this disorder (García-Sevilla et al., 1998). Other changes have been observed in animal models of Huntington's disease; we have previously shown that the I_2_ binding site is greatly reduced in Huntington's disease brains (Reynolds et al., 1996). Animal models suggest that the ATP synthesis is impaired in Huntington's diseases (Gines et al., 2003) and the administration of creatine increases the brain phosphocreatine level eliciting neuroprotective effects (Matthews et al., 1998). If this impairment of energy metabolism was due to a depletion of cellular energy stores, namely phosphocreatine, then it could be speculated that B-CK expression is altered in this disorder. In addition, it has been reported that the B-CK level is decreased in the brain of schizophrenics (Burbaeva et al., 2003). To date, the relationships between I_2_ sites and schizophrenia remain unknown; however, investigations into possible modulations in the density of I_2_ sites and/or possible effects of I_2_ ligands in this disorder would make interesting studies.

To what extent can the actions of I_2_ ligands be explained through an interaction with B-CK? B-CK is prominently expressed in neurons and it has been shown to influence membrane potentials and neurotransmitter release (Dunant et al., 1988). This could explain the mechanism by which I_2_ ligands may affect dopamine, 5-HT and noradrenaline release as well as their antidepressant-like actions in rats (Finn et al., 2003; Nutt et al., 1997). It has been reported that CK is directly and physically associated with the SUR2A subunit, but not the Kir6.2 subunit of the cardiac ATP sensitive potassium (K_ATP_) channels (Crawford et al., 2002). Also, there is evidence that CK regulates K_ATP_ channels in pancreatic β-cells (Krippeit-Drews et al., 2003) where B-CK is the major CK isoenzyme present. Interestingly, we know that some I_2_ and I_3_ ligands regulate K_ATP_ channels in pancreatic β-cells leading to insulin secretion (Morgan et al., 1999). CK is present in the hypothalamus including the arcuate nucleus (Ikeda and Tomonaga, 1988) where the I_2_ site is highly abundant (Lione et al., 1998), and a link between B-CK and K_ATP_ channels has been suggested to be important in the regulation of hypothalamic glucose-responsive (GR) neuronal activity. Hypothalamic GR neurons increase their firing rate with increased extracellular glucose levels and K_ATP_ channels in the hypothalamic GR neurons have been strongly suggested to be involved in the appetite control (Miki et al., 2001). Preliminary studies have shown that 2-BFI blocks K_ATP_ channels on hypothalamic GR neurons (personal communication with Professor M. Ashford, University of Dundee, U.K.). This may provide an explanation for the acute stimulation of eating induced by I_2_ ligands and the potassium sensitivity of I_2_ binding (Hudson et al., 2001). Undeniably, these hypotheses need to be examined thoroughly in future studies. In addition, further investigations should determine whether or not the changes in the level of I_2_ binding sites found in conditions such as schizophrenia, Huntington's disease, depression and opiate dependence are accompanied by similar alterations in B-CK expression, as seems to be the case in Alzheimer's disease.

In conclusion, we report the identity of a ∼ 45 kDa imidazoline binding protein to be brain creatine kinase and this enzyme possesses an I_2_ binding site. These striking findings will undoubtedly contribute to greater understanding of the effects of I_2_ ligands in psychiatric disorders and to developing such ligands as novel therapeutic drugs.

## 4 Experimental procedures

### 4.1 Materials

PharmaLink™ was purchased from Perbio Science, Cramlington, UK. Rabbit B-CK and protease inhibitors cocktail were purchased from Sigma, Poole, UK. Microcep™ and Nanoceps™ were purchased from Pall Life Sciences, Portsmouth, UK. Coomassie Brilliant Blue R-250 was obtained from Bio-Rad Laboratories, Hemel Hempstead, UK. 2-(2-benzofuranyl)2-imidazoline (2-BFI), 2-(4,5-dihydroimidaz-2-yl)quinoline (BU224) and 5-isothiocyanato-2-benzofuranyl-2-imidazoline (BU99006) were kindly synthesised by Dr S. M. Husbands, University of Bath, U.K. [^3^H]-2-BFI (specific activity of 70 Ci mmol^− 1^) was purchased from GE Healthcare, Amersham, U.K.

### 4.2 Isolation of ∼ 45 kDa I_2_ binding protein using a 2-BFI affinity column

New Zealand White rabbits of either sex (2.5–4 kg) were killed by a schedule 1 method (overdose of pentobarbitone, *i.v.*) and Wistar male rats (200–250 g) were killed by cervical dislocation. Whole brains were immediately removed and P_2_ membranes were prepared as described previously (Lione et al., 1996). These P_2_ membranes were solubilised in 0.5% CHAPS in the presence of protease inhibitors cocktail. The mixture was constantly stirred for 2 h on ice followed by centrifugation at 100,000 *g* for 30 min at 4 °C. A 2-BFI affinity column was synthesised using PharmaLink™. The PharmaLink™ gel (10 ml) was incubated with 20 mM 2-BFI in 0.1 M MES, pH 4.7, in the presence of formaldehyde (2.5%v/v final) for 40 h at 40 °C with constant agitation. The gel was then transferred into a column (2.5 × 10 cm) and unbound 2-BFI was washed off with 0.1 M Tris, pH 8.0 (240 ml). Affinity chromatography was performed similarly to previously described (Escribá et al., 1995; Wang et al., 1992). The 2-BFI affinity column was equilibrated with 0.05% CHAPS (50 ml) prior to the loading of the solubilised proteins (∼ 2 mg ml^− 1^, ∼ 17 ml). The column was then washed with 0.05% CHAPS and I_2_ binding proteins bound to the 2-BFI affinity column were eluted off with 20 mM idazoxan. The column was again washed with 0.05% CHAPS before all the residual proteins were removed by 1 M NaCl. The amount of proteins eluted was monitored by measuring their absorbance at 280 nm using a UV monitor (Model EM-1 Econo UV monitor, Bio-Rad Laboratories). The flow rate (1 ml min^− 1^) was maintained by a peristaltic pump. The fractions eluted with 20 mM idazoxan (6 ml) were collected and concentrated using Microcep™ by centrifugation at 5700 *g* for 90 min.

### 4.3 SDS-PAGE and electroblotting of I_2_ binding proteins onto PVDF membranes

SDS-PAGE (12%) was carried out according to the method of Laemmli (1970) and the gel was stained with Coomassie Blue. For protein blotting for the sequencer, previously described method was used with slight modifications (Dunbar and Wilson, 1994). Briefly, piperazine diacrylamide was used as a cross-linker and the gel was pre-run with 50 μM reduced glutathione for 1 h at 5 mA. The concentrated sample was mixed with a loading buffer (62.5 mM Tris–HCl (pH 6.8), 3% w/v SDS, 5% v/v mercaptoethanol, 25% v/v glycerol, and 0.05% w/v bromophenol blue) and heated for 10 min at 100 °C to denature the proteins. The final samples were loaded onto pre-run gel, which ran at 18 mA. Proteins were blotted onto PVDF membranes using Mini Trans-Blot^®^ Cell (Bio-Rad Laboratories) for 2 h at 300 mA.

### 4.4 N-terminal protein sequencing of isolated I_2_ binding protein

The ∼ 45 kDa band on PVDF membranes was excised then sequenced using Applied Biosystems Procise sequencer (Applied Biosystems, Warrington, U.K.), which utilises the classic Edman degradation procedure.

### 4.5 Radioligand binding assay

Rabbit B-CK (5 μg) or rabbit whole brain P_2_ membranes was incubated with 5 nM [^3^H]-2-BFI and 0.8 mg ml^− 1^ BSA for 30 min at 20 °C in the presence or absence of BU224 (10 μM) to establish specific binding. The reaction was terminated by the addition of polyethylene glycol (12.5% final). The samples were mixed well and precipitated proteins were separated from solution by centrifugation at 11,000 *g* for 10 min at 4 °C. The supernatant was discarded and the remaining pellets were briefly rinsed twice in ice-cold Tris–HCl buffer (50 mM Tris, 1 mM MgCl; pH 7.4). Scintillation fluid (12 ml) was added and the radioactivity remained in the samples was counted. For pre-treatment with the I_2_ irreversible ligand, the samples were incubated with BU99006 (10 μM) for 20 min at 37 °C followed by two washes in ice-cold Tris–HCl buffer by repeated centrifugation at 11,000 *g* for 5 min using Nanoceps™ before they were incubated with [^3^H]-2-BFI and subjected to the above procedures.

### 4.6 B-CK activity assay

The protocol used was modified from the methods described previously (Florini, 1989; Shainberg et al., 1971). The final composition of the assay mixture was 20 mM glucose, 10 mM magnesium acetate, 1 mM ADP, 10 mM AMP, 0.4 mM thio-NAD, 10 mM dithiothreitol, 0.5 U/ml hexokinase, 1 U/ml glucose-6-phosphate dehydrogenase and a range of phosphocreatine concentrations between 0 and 35 mM, all in 0.1 M glycylglycine, pH 6.75. Rabbit B-CK was pre-treated with various I_2_ ligands (1–20 μM) for 20 min at 37 °C before it was added to the assay mixture. The absorbance at 405 nm was measured for obtaining the initial velocities at 25 °C over 50 min using a 96 well plate reader. The results were analysed and graphs were created using GraphPad Prism 3.0.

### 4.7 Molecular modelling of the interaction between I_2_ ligands and B-CK crystal structure

The crystal structure (PDB: 1QH4) of chicken brain B-CK was utilised for the modelling studies. Hydrogens were added and minimised using the MMFF94 force field implemented within MOE (Chemical Computing Group, 2003.02) and keeping all other atoms fixed. 2-BFI and BU99006 were drawn using the Build option of MOE, keeping the imidazoline ring protonated and minimised using the MMFF94 force field.

2-BFI and BU99006 were docked to 1QH4 using GOLD (CCDC Software Ltd) with the GOLD Score Fitness Function. In addition, based on the reversibly docked structure with BU99006, we simulated covalent bond formation between Cys283 and the isothiocyanate group of BU99006 and the resulting modified protein was minimised using the MMFF94 forcefield. In this latter minimization step, only BU99006 and residues having an atom within 3 Angstroms of Cys283 were left flexible, all others were fixed.
